# Evaluation of Polymer Gel Electrolytes for Use in MnO_2_ Symmetric Flexible Electrochemical Supercapacitors

**DOI:** 10.3390/polym15163438

**Published:** 2023-08-17

**Authors:** Yu-Hao Lin, Wan-Tien Huang, Yi-Ting Huang, Yi-Ni Jhang, Tsung-Ting Shih, Murat Yılmaz, Ming-Jay Deng

**Affiliations:** 1 Department of Environmental Engineering, National Chung Hsing University, Taichung 40227, Taiwan; 11844@yahoo.com.tw; 2 Department of Applied Chemistry, Providence University, Taichung 43301, Taiwan; angel20020402@gmail.com (W.-T.H.); lilyacer11@gmail.com (Y.-T.H.); hi190523@gmail.com (Y.-N.J.); 3 Department of Chemistry, Fu Jen Catholic University, New Taipei City 24205, Taiwan; 142661@mail.fju.edu.tw; 4 Department of Chemical Engineering, Faculty of Engineering, Osmaniye Korkut Ata University, Osmaniye 80000, Turkey; muratyilmaz@osmaniye.edu.tr

**Keywords:** flexible, electrochemical supercapacitor, polymer gel electrolyte, deep eutectic solvent, Mn oxide

## Abstract

Flexible electrochemical supercapacitors (FESCs) are emerging as innovative energy storage systems, characterized by their stable performance, long cycle life, and portability/foldability. Crucial components of FESCs, such as electrodes and efficient electrolytes, have become the focus of extensive research. Herein, we examine deep eutectic solvent (DES)–based polymer gel systems for their cost-effective accessibility, simple synthesis, excellent biocompatibility, and exceptional thermal and electrochemical stability. We used a mixture a DES, LiClO_4_–2-Oxazolidinone as the electroactive species, and a polymer, either polyvinyl alcohol (PVA) or polyacrylamide (PAAM) as a redox additive/plasticizer. This combination facilitates a unique ion-transport process, enhancing the overall electrochemical performance of the polymer gel electrolyte. We manufactured and used LiClO_4_–2-Oxazolidinone (LO), polyvinyl alcohol–LiClO_4_–2-Oxazolidinone (PVA–LO), and polyacrylamide–LiClO_4_–2-Oxazolidinone (PAAM–LO) electrolytes to synthesize an MnO_2_ symmetric FESC. To evaluate their performance, we analyzed the MnO_2_ symmetric FESC using various electrolytes with cyclic voltammetry (CV), galvanostatic charge–discharge (GCD), and electrochemical impedance spectroscopy (EIS). The FESC featuring the PVA–LO electrolyte demonstrated superior electrochemical and mechanical performances. This solid-state MnO_2_ symmetric FESC exhibited a specific capacitance of 121.6 F/g within a potential window of 2.4 V. Due to the excellent ionic conductivity and the wide electrochemical operating voltage range of the PVA–LO electrolyte, a high energy density of 97.3 Wh/kg at 1200 W/kg, and a long-lasting energy storage system (89.7% capacitance retention after 5000 cycles of GCD at 2 A/g) are feasibly achieved. For practical applications, we employed the MnO_2_ symmetric FESCs with the PVA–LO electrolyte to power a digital watch and a light-emitting diode, further demonstrating their real-world utility.

## 1. Introduction

Electrochemical supercapacitors (ESCs) are considered as promising energy storage devices, attracting consdierable interest from both academia and industry. Their unique features, such as long cycle life, rapid charging/discharging, safety, and high power density [1,2,3,4,5], make them particularly appealing. However, compared to lithium-ion batteries, ESCs generally offer a lower energy density. In addition, the development of lithium-ion batteries in flexible electronic devices is constrained by their lack of flexibility and potential safety risks [1,2,3]. Presently, commercially available ESCs provide around 5–10 Wh/kg, insufficient to meet the high energy requirements of certain applications [6]. Thus, enhancing energy density has become a primary research focus for ESCs. The formula for energy density, E = 1/2CV^2^ [1,2], where C and V denote specific capacitance and operating voltage of ESCs, respectively, suggests that increasing V (or C) can effectively address the issue of low energy density. An essential factor determining operating voltage is the electrochemical potential window of electrolytes, which is restricted in aqueous electrolytes due to water splitting, limiting the electrochemical potential window to a mere 1.23 V [7,8]. Pan et al. structured a fiber ESC (>3 V) utilizing an ionic liquid–based electrolyte with a polymer gel electrolyte, resulting in improved energy density and flexibility [9]. Thus, creating high energy density ESCs is realized as a reliable choice for improving the operating voltage by way of manipulating the electrolytes or electrodes [9,10].

Organic electrolytes, regarded for their high electrolysis acceptance, are commonly used to construct high-voltage ESCs [11]. However, they present drawbacks such as low ionic conductivity, instability, flammability, safety hazards, and high costs, which impede their broader application [11]. Despite offering higher energy density, ESCs functioning in organic electrolytes have higher equivalent series resistance and lower ion-diffusion ability, adversely affecting power density [11,12]. This necessitates the exploration of alternative organic electrolytes.

Ionic liquids have garnered considerable attention from researchers owing to their impressive properties. They can serve multiple roles concurrently, which include conducting electrolytes, solvents, and polymeric host materials. Compared to water or standard organic solvents, ionic liquids possess superior attributes, such as high ionic conductivity, thermal stability, non-flammability, low volatility, and widespread electrochemical potential range (>4 V) [12,13]. Therefore, the development of polymer gel electrolytes based on ionic liquids is a promising avenue in energy and electrochemical research. Two main methods are employed to create ionic liquid–based polymer gel electrolytes. The first method involves direct addition of polymer into the ionic liquid to induce gelation, forming the electrolyte systems [13,14,15,16,17,18]. As part of this functionalized ionic liquids evolution, poly(ionic liquids) (or polymerized ionic liquids) have been investigated as an effective means to generate polymer gel electrolytes, owing to their amalgamation of polymer and ionic liquid benefits. The second method involves self-polymerization of functionalized ionic liquids to generate poly(ionic liquids)–based polymer gel electrolyte systems, which have shown utility in flexible electronic devices [19,20].

Although ESCs based on ionic liquids can support a higher electrochemical potential window [13,14], these ionic liquids typically require complex purification and production processes in a firmly measured environment to mitigate the risk of moisture contamination. Additionally, their high cost limits their application in ESCs. Therefore, more affordable electrolytes that do not require unique environments are promising. Deep eutectic solvents (DESs), a type of ionic liquid analogs, have been introduced for this purpose [21,22]. DESs used in electrochemical fields are generally mixtures of a quaternary ammonium salt and a hydrogen bond donor (usually an organic molecular component [22]). Hence, for the creation of cost effective and high energy density flexible electrochemical supercapacitors (FESCs), the development of an improved DES–based polymer gel electrolyte that can produce pseudocapacitance, achieve a large potential window, and exhibit excellent mechanical properties is crucial.

Herein, a neutral LiClO_4_–2-Oxazolidinone (LO) DES was chosen to achieve a widespread operating voltage and to produce capacitance. Additionally, polyvinyl alcohol (PVA), performing as a redox additive/plasticizer, was introduced to procedure the neutral redox reactive polyvinyl alcohol–LiClO_4_–2-Oxazolidinone (PVA–LO) polymer gel electrolyte. This electrolyte presents a unique ion-transport process and enhances the overall electrochemical performance. The PVA–LO electrolyte was further constructed with two identical flexible MnO_2_/carbon cloth (MnO_2_/CC) electrodes to form the MnO_2_ symmetric FESCs. The MnO_2_ symmetric FESC with a PVA–LO polymer gel electrolyte demonstrates outstanding electrochemical performance and mechanical properties. For comparison, polyacrylamide–LiClO_4_–2-Oxazolidinone (PAAM–LO) and LO were simultaneously manufactured and utilized for the construction of the MnO_2_ symmetric FESCs. Additionally, we also conducted in-situ X-ray absorption near-edge spectroscopy (XANES) studies to understand the energy storage mechanism and determine the changes in the oxidation state of the MnO_2_ electrodes with the PVA–LO polymer gel electrolyte during charge and discharge cycles. These investigations confirmed that the PVA–LO electrolyte can undergo redox reactions with the MnO_2_ electrodes, rather than exhibiting electric double layer behavior. The facile method used to configure the PVA–LO polymer gel electrolyte appears a simple and cheap approach for constructing FESCs with a broad operating voltage and outstanding overall performance.

## 2. Materials and Methods

### 2.1. Materials

The carbon cloth (CC) substrates were acquired from CeTech Co., (WOS 1010, Taichung, Taiwan). 2-Oxazolidinone (98%), LiClO_4_ (99%), Polyvinyl alcohol (PVA, Mw ~85,000), Polyacrylamide (PAAM, Mw ~150,000), Mn(CH_3_COOH)_2_∙4H_2_O, CH_3_CO_2_NH_4_, Ferrocene (99%), HNO_3_, Pt wire (99.9%) and Ag wire (99.9%) were all got from Sigma-Aldrich Chemical Co., (Taufkirchen, Germany) and employed without any purification.

### 2.2. Preparation of MnO_2_/CC Electrodes

The CC substrate was activated employing detergent, deionized water, 2 M HNO_3_, and deionized water. MnO_2_ nanofibers was prepared to make on the CC substrate with the electrochemical method; detailed information is presented in the Supplementary Materials [23,24]. The electrodeposited charge was 12 Coulomb or 3 C/cm^2^ (4.8 mg or 1.2 mg/cm^2^). Then, the MnO_2_/CC electrodes were completed. The mass of as-prepared samples was weighed by a microbalance (XP105DR, 0.01 mg resolution, Mettler Toledo, Switzerland).

### 2.3. Preparation of LO, PVA–LO, and PAAM–LO DES–Based Electrolzytes

The LO, PVA–LO, PAAM–LO electrolytes were made according to formerly published process [24,25]. The LO DES was obtained using a LiClO_4_/2-Oxazolidinone molar ratio of 1:4.2 [25,26]. The PVA (10 g) was dissolved in deionized water (100 mL) with agitating at 80 °C for 2 h (till the creation of a transparent PVA gel). 3 g of PAAM was dissolved in deionized water (150 mL) with agitating at 80 °C for 2 h (till the creation of a transparent PAAM gel). Then, the PVA–LO and PAAM–LO polymer gel electrolytes were achieved by mixing the 10 g of LO DES with the 10 g of PVA gel, and the 10 g of LO DES with the 10 g of PAAM gel heated at 110 °C in stirring till the uniform and transparent gel was achieved [24,25], respectively.

### 2.4. Assembly and Electrochemical Tests of MnO_2_ Symmetric Flexible Electrochemical Supercapacitors (FESCs)

The electrochemical potential windows of different DES–based systems were measured with cyclic voltammetry (CV) following a normal three-electrode system where glassy carbon electrode (CHI104, CH Instruments, Inc., Austin, TX, USA), an Ag electrode and a Pt wire were utilized as working, and counter electrodes soaked in the DES–based electrolyte, respectively. The Ag reference electrode was made by soaking an Ag wire in the LO DES–based solution containing 5 mM silver triflate separated by a glass frit [27]. It shows a potential of −0.25 V vs. Ag/AgCl (3 M KCl). After the test, the potential obtained through the three-electrode system was calibrated with respect to the ferrocene (Fc/Fc^+^) redox potential.

Preceding to the manufacture, every MnO_2_/CC electrode or filter paper (Kimwipe 34155, Kimberly-Clark Co., Neenah, WI, USA) were immersed into the LO, PVA–LO, or PAAM–LO electrolyte (2 h), respectively. The FESC was assembled operating a pair of MnO_2_/CC electrodes, a filter paper, and the different electrolyte (LO, PVA–LO, or PAAM–LO electrolyte) in a sandwich configuration. The filter paper was placed between two MnO_2_/CC electrodes. All electrochemical measurements of the glassy carbon electrode, MnO_2_/CC electrode, and FESC were approved at room temperature with potentiostat (Autolab, PGSTAT 128N, Utrecht, The Netherlands). The gravimetric capacitance (C_m,_ F/g) of MnO_2_ symmetric FESCs are acquired from the galvanostatic charge–discharge (GCD) profiles matching to the following [14,15,16]:

(1) $$C_{m}=\frac{I\;∆t}{m\;∆V\;}$$

where I means discharge current (A), Δt means discharge time (s), m denotes the weight of active material (g, contained in two electrodes), ΔV is the working voltage range (V). The energy density (E_m_, Wh/kg) and the power density (P_m_, Wh/kg) were measured corresponding to the following [14,15,16]:

(2) $$E_{m}=\frac{1000}{2\times 3600}\;C_{m}\;(∆V{)}^{2}$$

(3) $$P_{m}=3600\;\frac{\mathrm{Em}}{\;∆t\;}$$

where C_m_, ∆V, and ∆t is gravimetric capacitance (F/g), the working voltage of the FESC (V) and the discharge time (s).

### 2.5. Characterization

Scanning electron microscope (SEM, JEOL JSM-7610F, Tokyo, Japan) was used to obtain the surface morphology of the as-prepared samples. The valence states and chemical composition of the as-prepared products were studied by X-ray photoemission spectrometry (XPS, NSRRC BL 09A2, Hsinchu City, Taiwan). The ionic conductivity (σ) of different LO DES–based electrolytes was measured by electrochemical impedance spectroscopy (EIS). LO DES–based electrolytes were placed between two stainless steel sheets over the frequency range of 100 kHz to 0.1 Hz with 10 mV amplitude. The ionic conductivity (σ, S/cm) of electrolytes is determined by the following [14,28]:

(4) $$\sigma \;=\;\frac{l}{\;R\;\times \;A\;}\;$$

where *l* (cm) is the thickness of electrolytes between two steel sheets, R (Ω) is the bulk resistance of the electrolyte, A (cm^2^) is the area of the electrolyte contact with the steel sheets. The above EIS measurements were achieved by the electrochemical workstation (CHI 660E, CH Instruments, Inc., Austin, TX, USA). Viscosities were measured with a Brookfield viscometer (NDJ-1B, Ametek Brookfield, Middleborough, MA, USA). The variation of Mn oxidation states were estimated under changed working potentials in a FESC device with X-ray absorption near-edge spectroscopy (XANES) examined in the fluorescence yield mode (XANES, BL12B1 SPring-8 beamline, Tokyo, Japan) [15,16,23]. All X-ray absorption energies were standardized with the standard Mn mesh (6539 eV).

## 3. Results and Discussion

### 3.1. Characterization of MnO_2_/CC Electrode

**Figure 1:**
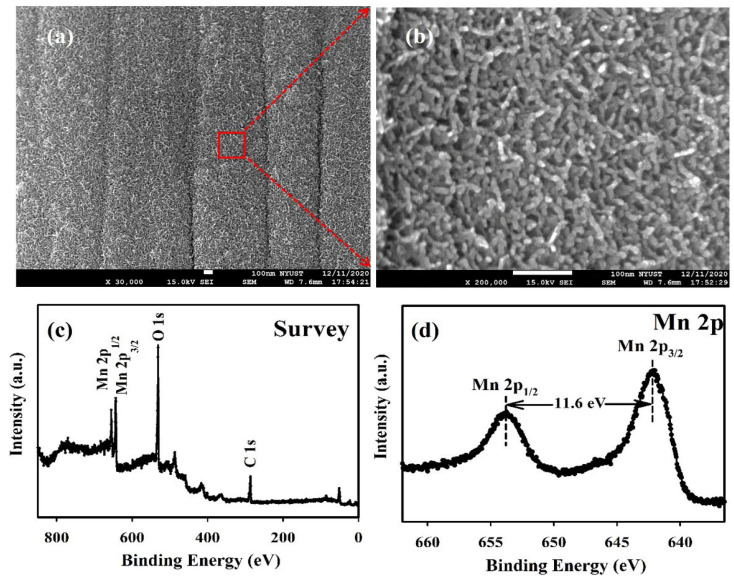
(**a**,**b**) SEM image of MnO_2_ nanofibers covered on the CC substrate. (**c**) XPS survey spectrum of as-prepared MnO_2_/CC electrode. (**d**) High-resolution Mn 2p of MnO_2_/CC electrode.

Figure 1a,b presents the SEM micrograph of the as-prepared MnO_2_/CC electrode. It is evident that the MnO_2_ nanofibers (approximately 15 nm in diameter) are stably and uniformly appended on the surface of the CC substrate. The unique porous structure of the MnO_2_ nanofibers ranges in length scales from the nanoscale to the scale of a few microns, enhancing the interaction area between MnO_2_ and the polymer gel electrolyte, thereby increasing the electrochemical redox reaction in the FESC. The chemical composition of the MnO_2_/CC electrode was characterized by XPS (Figure 1c,d). Figure 1c shows the XPS full survey spectrum, demonstrating the presence of Mn, O, and C elements in the electrode. Figure 1d depicts the typical Mn 2p spectrum of the MnO_2_/CC electrode, with two peaks at 653.7 eV for Mn 2p_1/2_ and 642.1 eV for Mn 2p_3/2_, a spin-energy separation of approximately 11.6 eV, indicating the primary presence of MnO_2_ in the as-prepared electrode. Figure S1a also presents further analysis of the metal oxidation state of MnO_2_/CC electrodes, where the Mn 2p_3/2_ peaks are fitted using Gaussian functions. The fitting binding energies of Mn 2p_3/2_ peaks at 641.6 and 642.6 eV correspond to Mn(III) and Mn(IV), respectively [29], indicating that Mn(IV) is dominant in the MnO_2_/CC electrode. As depicted in Figure S1b, the O 1s spectra of the MnO_2_/CC can be deconvoluted into three constituents representing different oxygen-containing species: anhydrous Mn oxide (Mn–O–Mn at 529.8 eV), Mn hydroxide (Mn–O–H at 531.2 eV), and structure water (H–O–H at 532.4 eV). This is consistent with previously reported MnO_2_ data [29,30,31]. Figure S2 also displays the X-ray diffraction (XRD) patterns of the MnO_2_/CC, and bare CC electrodes. The two smaller diffraction peaks at 37° (211) and 65.7° (002) are identified as α-type MnO_2_ (JCPDS 44-0141).

### 3.2. Electrochemical Performance of MnO_2_ Symmetric FESCs with Different LO DES–Based Electrolytes

The ionic conductivity of an electrolyte performs a key role in defining the electrochemical performances of ESCs, particularly for polymer gel electrolytes based ESCs. We determined the ionic conductivities of LO, PAAM–LO, and PVA–LO electrolytes (Table 1). Table 1 shows that the PVA–LO and PAAM–LO polymer gel electrolytes have higher viscosities than the LO electrolyte. For LO DES–based electrolytes, ionic conductivities increase when the same mass ratio of PVA gel (or PAAM gel) (1:1) is added, yielding maximum values of 30.6 mS/cm for the PVA–LO polymer gel electrolyte and 12.4 mS/cm for the PAAM–LO polymer gel electrolyte. This improvement in ion conductivities is likely due to the great plasticizing effect, which reduces the polymer chains, improving the flexibility of the polymer matrix, accelerating polymer segmental movement, and ion transport [13,28]. However, excessive or inappropriate addition of polymer gel leads to ion agglomeration, reducing ionic conductivities. The PVA–LO polymer gel electrolyte demonstrates greater ionic conductivity than the PAAM–LO polymer gel electrolyte, mainly due to its lower Coulomb interaction and hydrogen bond strength between anions and cations [13,28].

**Figure 2:**
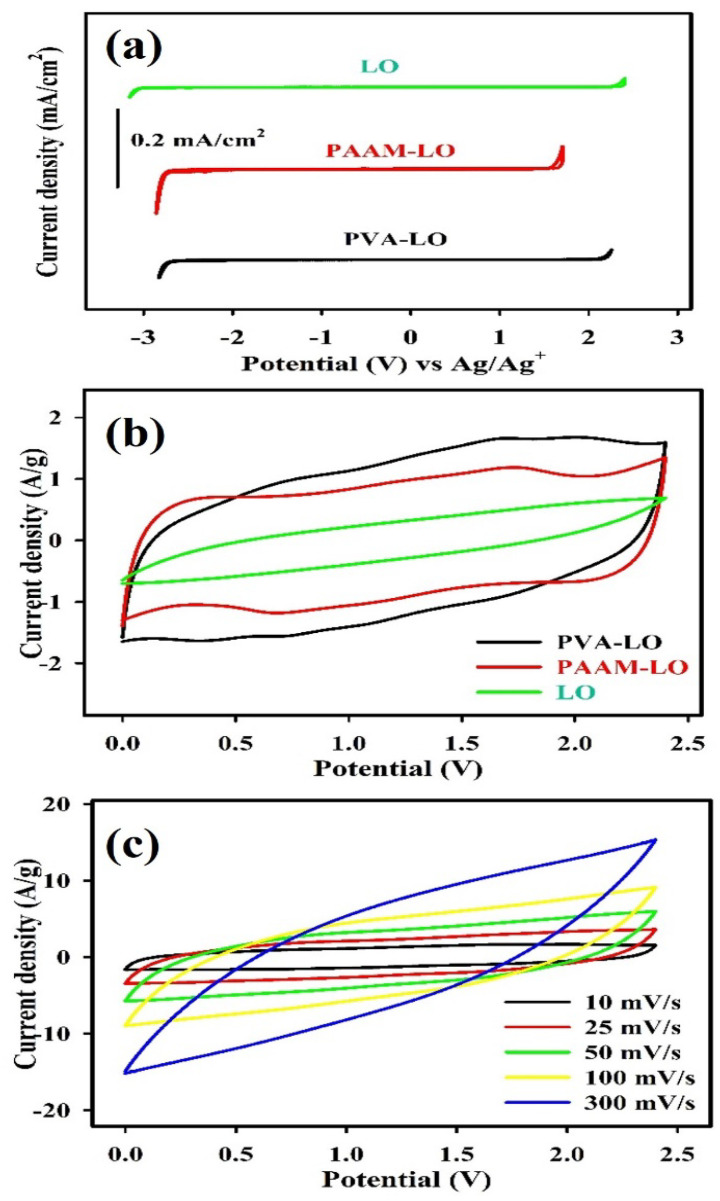
(**a**) CV curves of the glassy carbon electrode determined in PVA–LO, PAAM–LO, and LO electrolytes at 25 mV/s. (**b**) Electrochemical properties of MnO_2_ symmetric FESCs with PVA–LO, PAAM–LO, and LO electrolytes at 10 mV/s. (**c**) CV curves of MnO_2_ symmetric FESCs with PVA–LO polymer gel electrolyte at different scan rates.

Figure 2a appears the CV curves of a glassy carbon electrode determined in the PVA–LO, PAAM–LO, and LO electrolytes at 25 mV/s. The CV curves indicate that the electrochemical potential windows of the LO DES–based electrolytes exceed 3 V, demonstrating their potential as electrolytes for high operating voltage FESCs. The small current within the cathodic and anodic disintegration potentials could be associated with the disintegration of electrically active impurities in the LO DES–based electrolytes under the double-layer charging background. Figure 2b presents the CV curves of MnO_2_ symmetric FESCs with PVA–LO, PAAM–LO, and LO electrolytes at 10 mV/s. All MnO_2_ symmetric FESCs exhibit a quasi-rectangular CV profile and symmetric features, indicative of pseudocapacitive behavior, observable in a voltage range of nearly 2.4 V (from 0 to +2.4 V). This range is double that found in common aqueous electrolytes. The pseudocapacitive behavior is attributed to a swift sequence of reversible redox reactions between the Mn^3+^/Mn^4+^ ions from the MnO_2_ electrodes and the electrochemical adsorption/desorption of cations from the electrolyte occurring at the electrode/electrolyte interface [2]. Even though the CV curves of all MnO_2_ symmetric FESCs appear very similar, the area of the quasi-rectangular loops differs among the electrolytes. The MnO_2_ symmetric FESC with PVA–LO electrolyte shows better performance than those with PAAM–LO, and LO electrolytes. Among these, the MnO_2_ symmetric FESC with PVA–LO exhibits the largest area of the CV profile and the highest specific capacitance. Figure 2c appears the CV profiles of the MnO_2_ symmetric FESC with PVA–LO measured at various scan rates. The oxidation and reduction current densities increase with rising scan rates and maintain the quasi-rectangular loop. This suggests that the MnO_2_ symmetric FESC with PVA–LO can consistently exhibit pseudocapacitive behavior during the rapid ion transport and charge transfer processes at the electrode/electrolyte interface [2,28].

**Figure 3:**
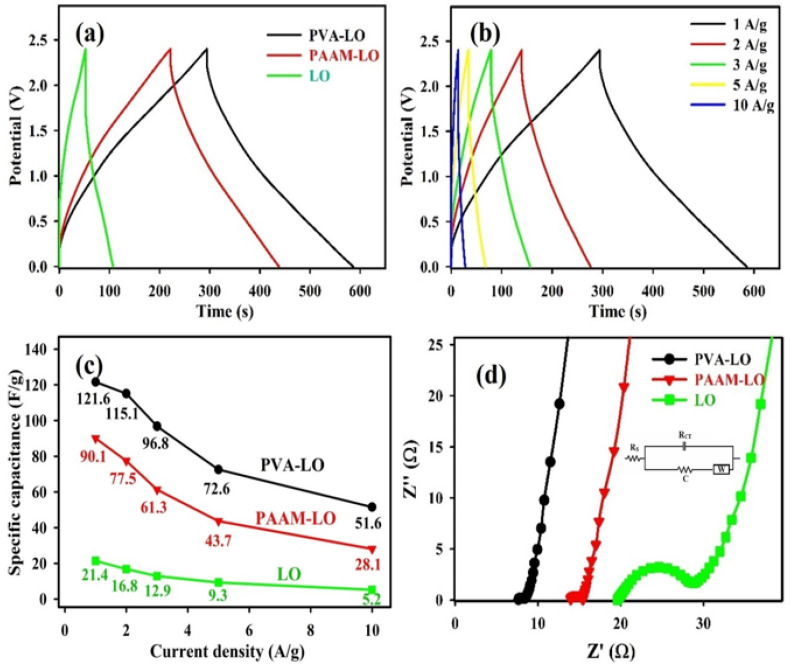
(**a**) GCD curves of MnO_2_ symmetric FESCs with PVA–LO, PAAM–LO, and LO electrolytes at a constant current density of 1 A/g. (**b**) GCD curves of MnO_2_ symmetric FESC with PVA–LO electrolyte at various current densities. (**c**) Gravimetric capacitances of MnO_2_ symmetric FESCs with PVA–LO, PAAM–LO, and LO electrolytes at various current densities. (**d**) Nyquist plots of MnO_2_ symmetric FESCs with PVA–LO, PAAM–LO, and LO electrolytes.

The GCD curves of the MnO_2_ symmetric FESC with PVA–LO, PAAM–LO, and LO electrolytes, analyzed at a constant current density (1 A/g), are proved in Figure 3a. From these GCD profiles, the C_m_ values of the MnO_2_ symmetric FESCs with PVA–LO, PAAM–LO, and LO electrolytes were determined, yielding results of 121.6, 90.1 and 21.4 F/g, respectively. The only variable between these MnO_2_ symmetric FESCs is the polymer gel used. Remarkably, the C_m_ of the MnO_2_ symmetric FESC with a PVA–LO polymer gel electrolyte is almost six times higher than that of the MnO_2_ symmetric FESCs with LO electrolyte. This difference may be due to two factors: (i) the superior ionic conductivity, and (ii) the adsorption of excessive counter-ions to the polymer chains, which facilitates ion transport and enhances the pseudocapacitive contribution from redox reactions at the electrode/electrolyte interface. GCD curves of the MnO_2_ symmetric FESC device with a PVA–LO polymer gel electrolyte at various current densities are proved in Figure 3b, which have a nearly symmetrical triangle shaped, denoting the appreciable electrochemical stability and reversible ion adsorption–desorption behavior at the electrode/electrolyte interface of the FESC device with a PVA–LO polymer gel electrolyte [32]. As the current density raises from 1 to 10 A/g, the C_m_ of the MnO_2_ symmetric FESCs with PVA–LO, PAAM–LO, and LO electrolytes decreases from 121.6 to 51.6 F/g, from 90.1 to 28.1 F/g, and from 21.4 to 5.2 F/g, respectively (Figure 3c). This is likely because the concentration polarization at the electrode/electrolyte interface and the ion-diffusion rate to the electrodes are not rapid enough to support the redox behavior, particularly at high current density, thus leading to a decrease in the reaction rate as well as in electric double-layer capacitance [28,33]. Electrochemical energy storage is primarily governed by either surface-controlled or diffusion-controlled processes. The current density of CV curves at low scan rates conforms to the following equation:

*i* = *av^b^* (5)

where *i* represents the response current and v stands for the scan rate, while coefficients *a* and *b* determine the behavior. A *b* value of 0.5 signifies diffusion-controlled electrochemical behavior, while *b* value of 1 indicates current dominance by the surface process, implying capacitive-controlled electrochemical behavior [34,35]. The calculated *b* value for the MnO_2_ symmetric FESC device with PVA–LO polymer gel electrolyte, based on Equation (5), is approximately 0.73. This result signifies that the current response is primarily capacitive-controlled behavior.

Next, we used EIS evaluations to assess the pseudocapacitive behaviors of these FESCs with different electrolytes. As presented in Figure 3d, the Nyquist plots of FESCs with PVA–LO, PAAM–LO, and LO electrolytes constitute a semi-circle at high frequency area and a line at low frequency area. The spectra are fitted to an equivalent circuit composed of an electrolyte resistance (R_S_), charge transfer resistance (R_CT_), Warburg element (W), and capacitor component (C) [36,37]. In the high frequency area, the intersection of the semi-circle with the x-axis of FESC with PVA–LO (R_CT_: 1.2 Ω) and FESC with PAAM–LO (R_CT_: 1.8 Ω) is smaller than that of FESC with LO (R_CT_: 8.9 Ω), indicating that mixing polymer species may significantly reduce the charge transfer resistance at the electrode/electrolyte interface. Starting from the intermediate area, the impedance diagram progressively transitions from a semi-circle to a straight line. In the low frequency area, the impedance diagram is fully characterized by a straight line, indicating a gradual shift in the control step of the process from an electrochemical step to a diffusion step. The slopes of the straight lines in the low-frequency area for FESC with PVA–LO and FESC with PAAM–LO are larger than that of FESC with LO. Additionally, FESC with PVA–LO exhibits the most vertical curve, indicating that it possesses ideal FESC characteristics. [36,37]. Among all FESCs with different electrolytes shown in Figure 3d, the FESC with PVA–LO exhibits the lowest charge transfer resistance and a lower Warburg component, contributing to its excellent supercapacitor performance. This observation further supports the notion that the lowest charge transfer resistance is attributed to the higher ionic conductivity in the PVA–LO polymer gel electrolyte, which facilitates the adsorption and desorption of electrolyte ions.

**Figure 4:**
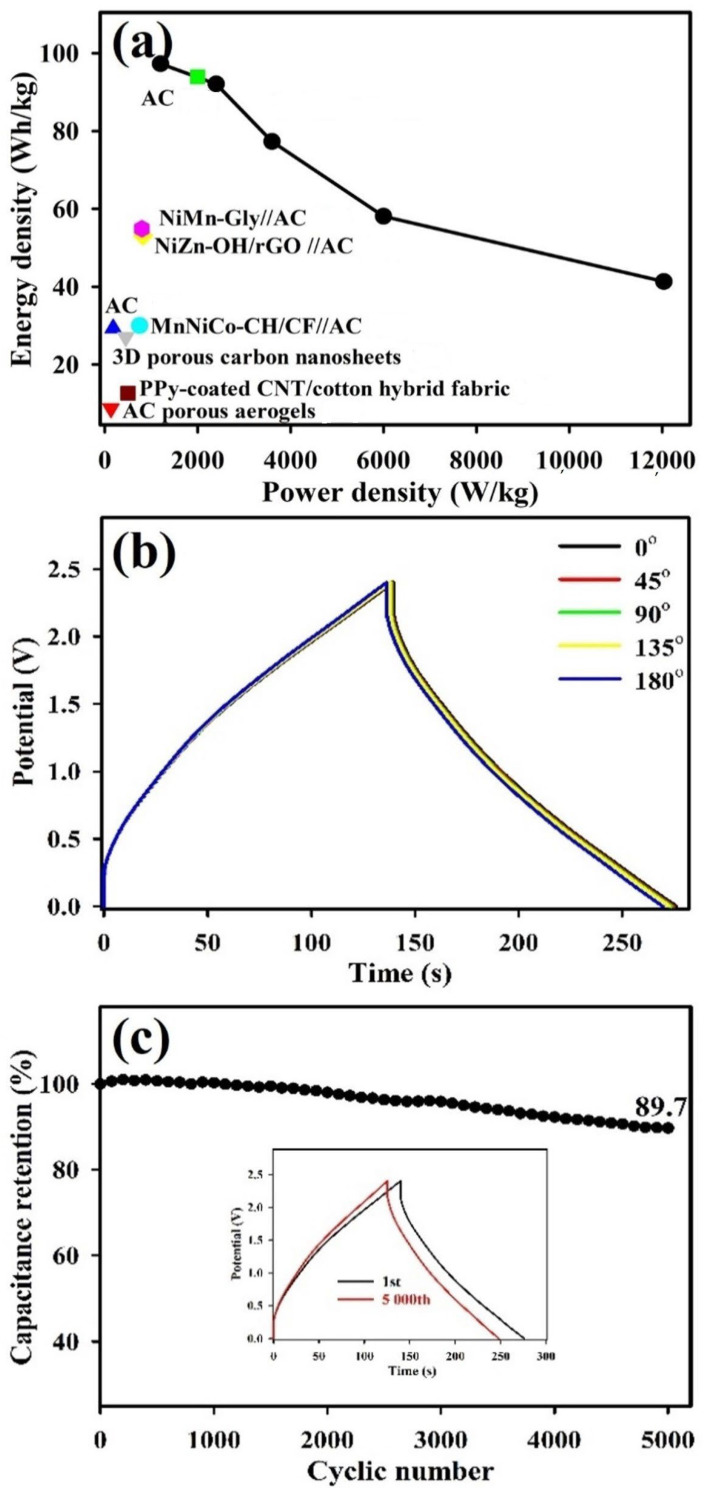
(**a**) Ragone plots of solid-state FESCs, (b) GCD cycles at a constant current density of 2 A/g under various bending angles (0°, 45°, 90°, 135°, 180°) and (**c**) Cycling performance at 2 A/g of MnO_2_ symmetric FESC device with PVA–LO polymer gel electrolyte [14,28,38,40,43,44,45,46].

Based on the GCD profiles obtained from different current densities (Figure 3c), the E_m_ and P_m_ of all FESCs are outlined and compared with other previously reported solid-state ESCs via Ragone plots to further understand the performance characteristics [14,28,38,39,40,41,42,43,44,45,46], as presented in Figure 4a. The energy densities of FESCs with PVA–LO, PAAM–LO, and LO electrolytes can reach 97.3, 72.1, and 17.1 Wh/kg, respectively. Remarkably, the energy density of the FESC with PVA–LO (97.3 Wh/kg) is almost six times higher than that of the FESCs with LO (17.1 Wh/kg). This surpasses the performance of solid-state ESCs with polymer gel electrolytes reported previously [14,28,38,39,40,41,42,43,44,45,46], making it a promising candidate for energy storage system applications. When compared with different electrode materials and polymer gel electrolytes, the FESCs developed with PVA–LO show superior performance, as detailed in Table 2. This exceptional energy density can be attributed to the wide operating voltage obtained from the LO DES system and the additional pseudocapacitance provided by the adsorption of excessive counter-ions to the polymer chains. This assists ion-transport and improves the pseudocapacitive contribution from redox reactions at the electrode/electrolyte interface.

Figure 4b presents the GCD profiles of the developed FESC with PVA–LO under various bending angles. The C_m_ of the FESC device with PVA–LO was calculated based on the GCD curves at 2 A/g, yielding C_m_ values of 115.1, 114.5, 114.1, 113.7, and 112.4 F/g under different bending angles (0°, 45°, 90°, 135°, 180°). To evaluate the cycling stability, the GCD cycles at 1 A/g were analyzed over 5000 cycles. Figure 4c reveals that the capacitance retention of the FESC with the PVA–LO polymer gel electrolyte initially increases and subsequently decreases with cycling, an attribute that owes itself to the activation process of the electrode material in the electrolyte. This decrease in capacitance retention is attributed to the enhanced aggregation of LO DES in the pores of the MnO_2_ nanofibers electrode and the reduction in the redox activity of the redox process [28]. After 5000 cycles, the FESC device with the PVA–LO polymer gel electrolyte demonstrated a capacitance retention of 89.7%, indicating excellent cyclic durability. Furthermore, the GCD cycles for the 1st cycle and the 5000th cycle were depicted in the inset of Figure 4c. Evidently, the charge and discharge curves exhibited remarkable similarity, suggesting excellent cycling stability over time. The promising cyclic stability and high charge–discharge reversibility of this FESC indicate its practicality for long-term usage. Figure S3 displays the SEM micrographs of the MnO_2_/CC electrode after undergoing 5000 GCD cycles with the PVA–LO polymer gel electrolyte. As observed, the nanofiber surface morphology retained its integrity after cycling, with almost no structural changes observed. This confirms the durability of the MnO_2_/CC electrode operated with the PVA–LO polymer gel electrolyte. 

**Figure 5:**
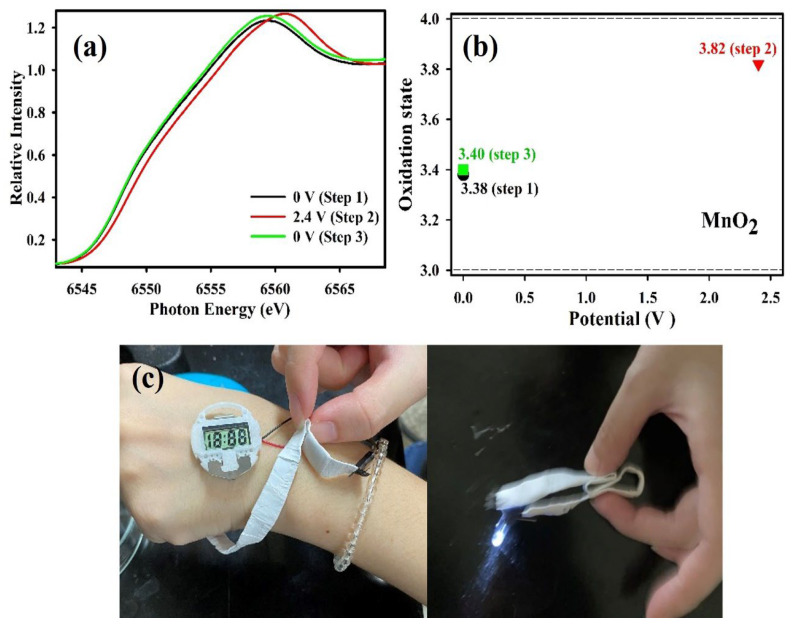
(**a**) Mn K-edge XANES spectra of MnO_2_ symmetric FESC device with PVA–LO polymer gel electrolyte under different operating voltages (0 V, 2.4 V, 0 V). (**b**) Varies of Mn oxidation states (MnO_2_ symmetric FESC device) under different operating voltages. (**c**) Photograph of MnO_2_ symmetric FESC device with PVA–LO polymer gel electrolyte in series with outstanding flexibility/twistability and presentations the digital watch and LED operated by the devices.

To elucidate the pseudocapacitive performance and changes in the Mn oxidation state during the GCD cycles of the developed FESC device with PVA–LO polymer gel electrolyte, in-situ XANES studies were conducted. These were aimed at identifying the shifts in chemical states under varying applied voltages. Before initiating the XANES studies, the working potential was maintained at 2.4 V for 15 min. Figure 5a illustrates the in-situ XANES curves of the MnO_2_ symmetric FESC with PVA–LO, examined under three different voltages: 0 V, 2.4 V, and backtrack to 0 V. The Mn K-edge XANES curves of the FESC device with PVA–LO displayed an energy increase as the applied voltage rose, returning to its initial position when the potential was reverted. The E_0_ (threshold energy) derived from the XANES curves in Figure 5a is associated with the oxidation states of Mn [47,48]. Figure 5b showcases the average oxidation state (Mn) of the MnO_2_/CC electrode in the FESC device with the PVA–LO polymer gel electrolyte. Standard samples of Mn, Mn_2_O_3_, and MnO_2_ were analyzed. The Mn oxidation state, estimated from the XANES curves of the MnO_2_/CC electrode, verifies that the modification of the oxidation state (Mn) from 0 to 2.4 V is approximately 0.42. The Mn valence changes of the MnO_2_ symmetric FESC with PAAM–LO (0.31) and LO (0.1) electrolyte were observed to be within the range of 0 V to +2.4 V (Figure S4), indicating a smaller magnitude of change compared to the MnO_2_ symmetric FESC with PVA–LO polymer gel electrolyte. This observation strongly indicates the exceptional ionic and electronic conductivity of the MnO_2_ symmetric FESC with PVA–LO polymer gel electrolyte. The continuous and reversible Mn^3+^/Mn^4+^ reaction facilitated by PVA–LO contributes to the superior performance of the MnO_2_ symmetric FESC. The valid redox reaction brought the outstanding energy density of 97.3 Wh/kg at the power density of 1200 W/kg for the FESC device. This also implies that the adsorption of excessive counter-ions to the polymer chains aids ion-transport and enhances redox kinetics at the electrode/electrolyte interfaces. Lastly, we connected the MnO_2_ symmetric FESC device with PVA–LO in series (Figure 5c), successfully powering a digital watch and light-emitting diode. These results strongly endorse our claim that the MnO_2_ symmetric FESC device with PVA–LO polymer gel electrolyte exhibits superior performance in flexible energy storage applications. Based on the findings from EIS, GCD, and XANES analyses, it is evident that the PVA–LO polymer gel electrolyte not only enhances conductivity and mitigates volume changes but also widens the working potential window and facilitates cation insertion/extraction. In summary, this study presents a simple design utilizing a neutral polymer gel electrolyte, enabling the attainment of high operating voltages and the fabrication of stable symmetric MnO_2_ solid-state FESC devices.

## 4. Conclusions

In this study, we successfully manufactured a neutral redox-active PVA–LO polymer gel electrolyte. PVA serves a dual role as both a redox additive/plasticizer and a facilitator of a unique ion-transport process, enhancing the overall pseudocapacitive contribution through reversible redox reactions at the electrode/electrolyte interfaces. The MnO_2_ symmetric FESC device with PVA–LO demonstrated exceptional electrochemical performance, achieving a broad working voltage of 2.4 V and a high specific capacitance (121.6 F/g). Due to the advantageous broad operating voltage and great C_m_, the MnO_2_ symmetric FESC device with PVA–LO polymer gel electrolyte offered an impressive energy density of 97.3 W h/kg, nearly six times greater than the FESC device with LO (17.1 Wh/kg). Additionally, it showcased remarkable cyclic stability, retaining 89.7% of its capacitance after 5000 cycles, and demonstrated excellent mechanical performance, surpassing commonly reported solid-state ESCs. In conclusion, the facile method used to configure the electrolyte seems to be a simple and cost-effective approach for constructing FESCs with a broad operating voltage and outstanding overall performance.

## Figures and Tables

**Table 1:** Physical data of the three DES–based electrolytes applied in this study.

Electrolyte	Viscosity (cp)	Conductivity (mS/cm)	Ref.
LO (1:4.5) ^a^	X	0.7	[26]
LO (1:4.2) ^a^	90.9	1.1	This work
PAAM–LO	217.2	12.4	This work
PVA–LO	798.2	30.6	This work

^a^ Molar ratio.

**Table 2:** Comparison of solid-state FESCs.

Electrode Material	Electrolyte	Potential Windows (V)	Energy Density (Wh/Kg)	**Ref.**
AC porous aerogels	PEO–LiTFSI– VBIMBr	2.0	8.7	[14]
AC	PVA–Li_2_SO_4_–BMIMI	1.5	29.3	[28]
AC	PUA–EMITFSI	4.0	93.9	[38]
activated CF	Agar/PVA–Li_2_SO_4_–EMIMBF_4_	1.0	4.0	[39]
3D porous carbon nanosheets	PVA–Na_2_SO_4_	1.8	27.0	[40]
PEDOT:PSS–rGO	UPyHCBA–acrylamide hydrogel	0.6	1.9	[41]
porous graphitic carbon	PVA–KOH	1.0	18.0	[42]
PPy-coated CNT/cottonhybrid fabric	PVA–H_2_SO_4_	0.8	12.6	[43]
NiZn–OH/rGO//AC	PVA–KOH	1.6	53.7	[44]
NiMn–Gly//AC	PVA–KOH	1.6	54.4	[45]
MnNiCo–CH/CF//AC	PVA–KOH	1.5	30.4	[46]
MnO_2_/CC//MnO_2_/CC	LO	2.4	17.1	This work
MnO_2_/CC//MnO_2_/CC	PAAM–LO	2.4	72.1	This work
MnO_2_/CC//MnO_2_/CC	PVA–LO	2.4	97.3	This work

## Data Availability

Data is contained within the article.

## Supplementary Materials

The following are available online at https://www.mdpi.com/article/10.3390/polym15163438/s1, Figures S1–S4: Evaluation of polymer gel electrolytes for use in MnO_2_ symmetric flexible electrochemical supercapacitors.
