# Supplementary material for: Evaluation of Polymer Gel Electrolytes for Use in MnO2 Symmetric Flexible Electrochemical Supercapacitors
Source: Polymers (Basel). 2023 Aug 17;15(16):3438. doi: 10.3390/polym15163438 (PMC10458581; doi:10.3390/polym15163438)
Supplement: Supplementary file 1 [file polymers-15-03438-s001.zip › polymers-2551651-supplementary.pdf]

polymers

MDPI

Article

Supplementary Materials: Evaluation of Polymer Gel Electrolytes for Use in MnO_2 Symmetric Flexible Electrochemical Supercapacitors

Yu-Hao Lin [1], Wan-Tien Huang [2], Yi-Ting Huang [2], Yi-Ni Jhang [2], Tsung-Ting Shih [3], Murat Yılmaz [4] and Ming-Jay Deng [2,*]

[1] Department of Environmental Engineering, National Chung Hsing University, Taichung 40227, Taiwan; 11844@yahoo.com.tw

[2] Department of Applied Chemistry, Providence University, Taichung 43301, Taiwan; angel20020402@gmail.com (W.-T.H.); lilyacer11@gmail.com (Y.-T.H.); hi190523@gmail.com (Y.-N.J.)

[3] Department of Chemistry, Fu Jen Catholic University, New Taipei City 24205, Taiwan; 142661@mail.fju.edu.tw

[4] Department of Chemical Engineering, Faculty of Engineering, Osmaniye Korkut Ata University, Osmaniye 80000, Turkey; muratyilmaz@osmaniye.edu.tr

* Correspondence: dengmj1020@pu.edu.tw

Citation: Lin, Y.-H.; Huang, W.-T.; Huang, Y.-T.; Jhang, Y.-N.; Shih, T.-T.; Yılmaz, M.; Deng, M.-J. Evaluation of Polymer Gel Electrolytes for Use in MnO_2 Symmetric Flexible Electrochemical Supercapacitors. *Polymers* **2023**, *15*, 3438. https://doi.org/10.3390/polym15163438

Academic Editor: Jeong In Han

Received: 26 July 2023

Revised: 11 August 2023

Accepted: 15 August 2023

Published: 17 August 2023

Copyright: © 2023 by the authors. Licensee MDPI, Basel, Switzerland. This article is an open access article distributed under the terms and conditions of the Creative Commons Attribution (CC BY) license (https://creativecommons.org/licenses/by/4.0/).

Preparation of MnO_2/CC electrodes

The CC substrate, a cleaned film with an area of approximately 6 cm², underwent pretreatment steps, including cleaning in deionized water for 2 minutes, immersion in 2 M HNO_3 for 10 minutes, rinsing with deionized water, and finally drying in a fume cupboard at room temperature. The deposition process was conducted at room temperature in a three-electrode cell with a Pt wire counter electrode and a saturated calomel reference electrode (SCE, CHI150, CH Instruments, Inc., USA) on an Autolab PGSTAT 128N potentiostat. A CC substrate with an area of approximately 4 cm² was used as the working electrode. The MnO_2 nanofiber layers were electrodeposited onto the CC substrate at 0.45 V using a solution containing 0.3 M $Mn(CH_3COO)_2$ and 0.1 M $CH_3CO_2NH_4$ at room temperature. This process resulted in the assembly of the nanostructured MnO_2/CC electrode, with a Pt wire counter electrode and a SCE as the reference electrode. Then, the MnO_2/CC electrodes were completed. The typical mass of the deposited MnO_2 (12 Coulomb, 3 C/cm²), measured with a microbalance (XP105DR Mettler Toledo, accuracy 0.1 mg), was 4.8 mg (equivalent to 1.2 mg/cm² for the deposited area). The mass of dead components such as the electrolyte, CC, and separator (Kimwipe 34155, Kimberly-Clark Co., USA), was not considered as part of the active materials (one MnO_2 electrode with 4.8 mg or 1.2 mg/cm²).

Electrochemical measurements:

The electrochemical potential windows of different DES–based systems were measured with cyclic voltammetry (CV) following a normal three-electrode system where a glassy carbon electrode (CHI104, CH Instruments, Inc., USA), an Ag electrode, and a Pt wire were utilized as working, and counter electrodes soaked in the DES–based electrolyte, respectively. The Ag reference electrode was made by soaking an Ag wire in the LO DES–based solution containing 5 mM silver triflate separated by a glass frit [**27**]. It shows a potential of – 0.25 V vs. Ag/AgCl (3M KCl). After the test, the potential obtained through the three-electrode system was calibrated with respect to the ferrocene (Fc/Fc^+) redox potential.

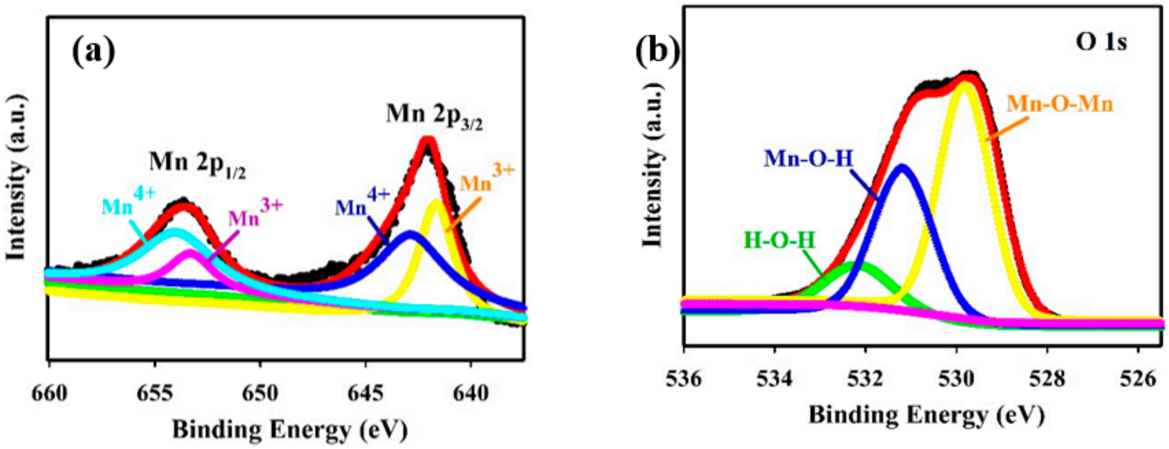

Figure S1. XPS spectra of (a) Mn 2p, and (b) O 1s of the as-prepared MnO_2/CC electrode.

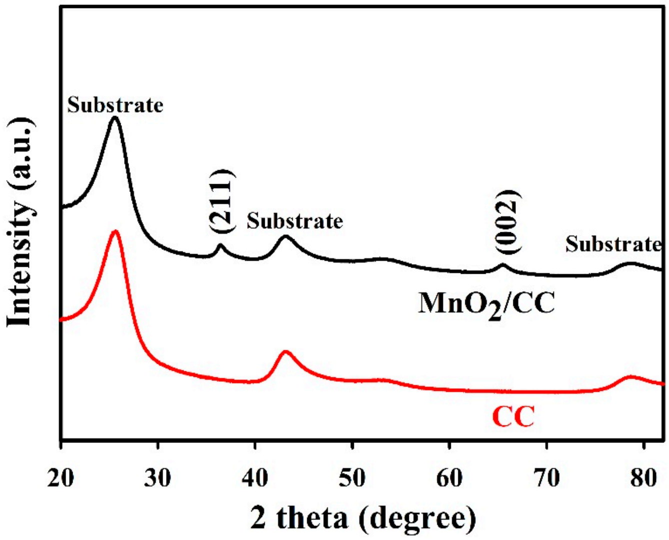

Figure S2. XRD patterns of MnO_2/CC, and bare CC electrodes, respectively.

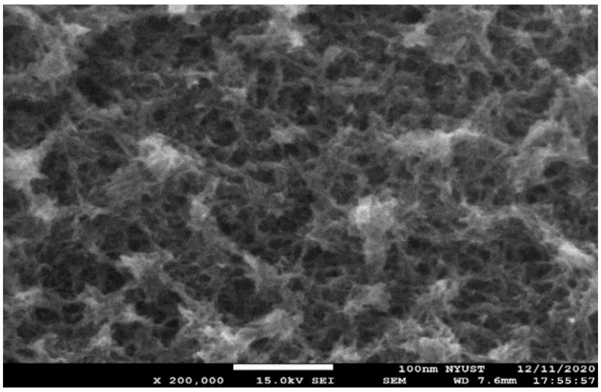

Figure S3. SEM micrographs of the MnO_2/CC electrode after undergoing 5,000 GCD cycles with the PVA–LO polymer gel electrolyte.

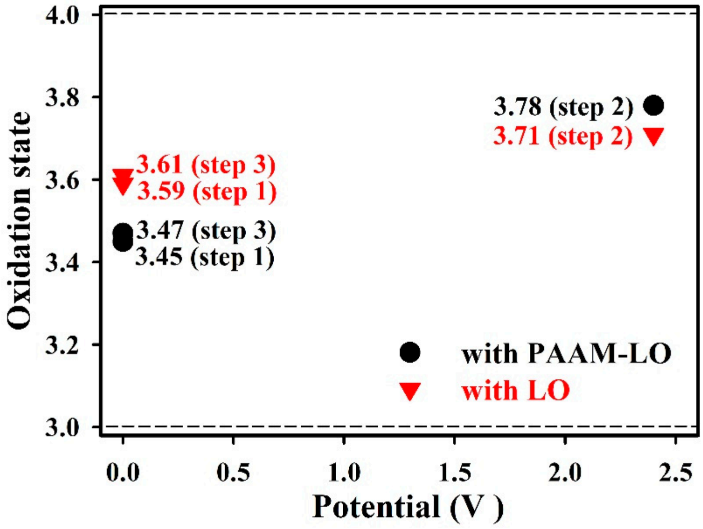

Figure S4. (c) Varies of Mn oxidation states (MnO_2 symmetric FESC device PAAM–LO, and LO electrolytes) under different operating voltages.

Disclaimer/Publisher's Note: The statements, opinions and data contained in all publications are solely those of the individual author(s) and contributor(s) and not of MDPI and/or the editor(s). MDPI and/or the editor(s) disclaim responsibility for any injury to people or property resulting from any ideas, methods, instructions or products referred to in the content.
